# Factors Affecting Aggregate Formation in Cell Models of Huntington’s Disease and Amyotrophic Lateral Sclerosis

**Published:** 2013

**Authors:** V. F. Lazarev, D. V. Sverchinskyi, M. V. Ippolitova, A. V. Stepanova, I. V. Guzhova, B. A. Margulis

**Affiliations:** Institute of Cytology, Russian Academy of Sciences, Tikhoretsky ave., 4, St. Petersburg, 194064

**Keywords:** neurodegenerative pathologies, glyceralgehyde-3-phosphate dehydrogenase, chaperones, mutant proteins, aggregation

## Abstract

Most neurodegenerative pathologies stem from the formation of aggregates of mutant proteins, causing dysfunction and ultimately neuronal death. This study was aimed at elucidating the role of the protein factors that promote aggregate formation or prevent the process, respectively, glyceraldehyde-3-dehydrogenase (GAPDH) and tissue transglutaminase (tTG) and Hsp70 molecular chaperone. The siRNA technology was used to show that the inhibition of GAPDH expression leads to a 45–50% reduction in the aggregation of mutant huntingtin, with a repeat of 103 glutamine residues in a model of Huntington’s disease (HD). Similarly, the blockage of GAPDH synthesis was found for the first time to reduce the degree of aggregation of mutant superoxide dismutase 1 (G93A) in a model of amyotrophic lateral sclerosis (ALS). The treatment of cells that imitate HD and ALS with a pharmacological GAPDH inhibitor, hydroxynonenal, was also shown to reduce the amount of the aggregating material in both disease models. Tissue transglutaminase is another factor that promotes the aggregation of mutant proteins; the inhibition of its activity with cystamine was found to prevent aggregate formation of mutant huntingtin and SOD1. In order to explore the protective function of Hsp70 in the control of the aggregation of mutant huntingtin, a cell model with inducible expression of the chaperone was used. The amount and size of polyglutamine aggregates were reduced by increasing the intracellular content of Hsp70. Thus, pharmacological regulation of the function of three proteins, GAPDH, tTG, and Hsp70, can affect the pathogenesis of two significant neurodegenerative diseases.

## INTRODUCTION

Progressive neuronal death in certain parts of the brain is the culprit in most neurodegenerative disorders. The development of these pathologies starts from intra- (Parkinson’s and Huntington’s diseases) or extracellular accumulation (Alzheimer’s disease) of the aggregates of mutant proteins or their oligomers [1]. These structures are toxic for brain cells; they cause immediate neuronal death, although there is some evidence that they can exist in neurons for dozens of years and turn into an active toxic factor only at some moment in time [2].

There are two hypotheses for the aggregate formation of mutant proteins. According to one, the aggregates can form due to the formation of hydrogen bonds between the β-sheets of a damaged or a mutant protein molecule [3]. These structures are inaccessible to strong dissociating solvents, in particular to sodium dodecylsulfate (SDS). The high density of the aggregating material presumably prevents the cell from using proteolytic systems, proteasomes, and phagosomes to fight the aggregates [4]. According to the second hypothesis, amyloid aggregates can form due to covalent cross-links between mutant protein molecules and other cell proteins. The formation of such cross-links is typical of the so-called polyglutamine pathologies, which are based on mu tations resulting in the formation of anomalously long glutamine repeats in huntingtin molecules, various ataxins, and the androgen receptor [5–7]. Polyglutamine repeats can also be deleted in the reaction catalyzed by tissue transglutaminase (tTG) and covalently bind to donors of lysine ε-amino groups. The hypothesis about the key role of tTG in the formation of insoluble aggregates of mutant huntingtin is supported by data that indicate that the polyglutamine domains of huntingtin act as the active substrate for this enzyme; aggregation almost stops in the absence of tTG [8]. The glycolytic enzyme glyceraldehyde-3- phosphate-dehydrogenase (GAPDH) can be used as a lysine donor in the reaction catalyzed by tTG [9]. We recently showed that GAPDH is indeed capable of forming aggregates with mutant huntingtin [10]. The data regarding the detection of GAPDH in aggregates or deposits of other mutant proteins (e.g., amyloid precursor and α-synuclein) [11, 12] provide grounds to regard GAPDH as a certain universal substrate for the aggregation processes [13]. One of the aims of our study was to determine whether GAPDH and tTG can not only participate in the aggregation of mutant huntingtin, but also contribute to the pathogenesis of a completely different disease, amyotrophic lateral sclerosis (ALS).

ALS is one of the most common neurodegenerative disorders, which manifests itself in the degeneration of neurons in the spinal cord, brain stem, and cortex [14]. ABS is inherited in approximately 14% of all cases; among those, 20% are caused by mutations in the superoxide dismutase 1 (SOD1) gene. Mutations occur in all exons of the *SOD1 *gene; some of them result in folding disturbance and protein product aggregation. These mutations include the replacement of glycine 93 by alanine, G93A [15]. Thus, the first section of our study is devoted to the analysis of the functions of GAPDH and tTG in cells simulating HD and ALS.

In addition to the proteins involved in the formation of cytotoxic oligomers and aggregates, the cell contains factors that impede this process. First of all, these factors include molecular chaperons (in particular, proteins that belong to the Hsp70 family and the co-chaperons Hdj1/2) [16, 17]. An enhanced expression of the genes of these factors in the cell or in a transgenic organism inhibits the aggregation and reduces pathogenic symptoms [18]. It is believed that during the initial stages of aggregate formation chaperon Hsp70 binds their components, thus inhibiting the aggregation process [10, 19, 20]. The features of Hsp70 functioning during the aggregation of mutant huntingtin in a cell culture model of HD are considered in the second section of our study.

## EXPERIMENTAL

**Cells**

Human neuroblastoma cell lines (SK-N-SH and SH-SY-5Y) were provided by D. R ubinztein (Cambridge University, UK); neuronal embryonic cells HNSC3148 were provided by L.I. Korochkin (Institute of Gene Biology, Russian Academy of Sciences, Moscow, Russia). The cloned cell line SK-N-SH/hsp70 had been obtained at the Laboratory of Cell Protection Mechanisms (Institute of Cytology, Russian Academy of Sciences) via cell transfection with a plasmid with an inserted *hsp70 *gene under the control of the metallothionein promoter [10, 21]. The cells were grown in a DMEM medium (Biolot, St. Petersburg, Russia) with addition of *L*-glutamine, a 10% fetal bovine serum, and 50 mg/ml gentamicin (PanEco, Moscow, Russia) at 37°С in a 5% СО_2_ atmosphere. SK-N-SH/hsp70 cells were grown in the presence of 100 μM genecitin.

**Cell transfection**

Plasmids containing exon 1 of the gene encoding huntigtin with a normal (Q25) and pathogenic (Q103) number of glutamine residues (hereinafter referred to as the *Q25 *and *Q103 *genes, respectively) linked to the *EGFP *gene encoding an enhanced green fluorescent protein (plasmids were provided by D. R ubinztein) and plasmids containing the wild-type superoxide dismutase 1 (SOD1_wt_) gene and the mutant variant SOD_1G93A_ linked to the EGFP gene (provided by M. Cheetham, University College London, UK) were used in this study. Small interfering RN A (siRN A) specific to GAPDH was purchased from Ambion (Ambion/Life Technology/Invitrogen, USA).

The cells were seeded into the wells of a 24- or 6-well plate 24 h prior to the transfection at a concentration of 3×10^5^ cells/ml. Transfection was performed using the Lipofectamine–PLUS reagent (Invitrogen, USA) according to the manufacturer’s recommendations.

The cells were transfected with *GAPDH *siRN A 24 h prior to transfection with the *Q103 *and *Q25 *genes or simultaneously with transfection with the *SOD1_G93A_*or* SOD1_WT_*gene.

**Confocal microscopy**

The cells were seeded onto glass coverslips placed into the wells of a 24-well plate at a concentration of 3× 105 cells/ml. In order to determine the colocalization of GAPDH and tTG with Q103, 48 h after the transfection, the cells were fixed in 4% formaldehyde (Sigma, USA) in a phosphate buffer saline (PBS) for 30 min, washed with pure PBS, and permeabilized with cold 96% ethanol for 5 min at –20°С. The cells were incubated with specific anti-GAPDH (Abcam, UK) or anti-tTG antibodies (Sigma, USA) overnight. After the cells had been washed in PBS, they were incubated with secondary antibodies conjugated to a CY3 fluorescent label (JacksonLab., USA). The specimens were studied using a Leica TC S SL confocal microscope (Germany). The sequential scan mode was used to avoid nonspecific interference from fluorochromes. The aggregate size was determined using a LSM510 Zeiss confocal microscope and the Zeiss LSM Image Examiner software, version 2.80.1123 (Сarl Zeiss, Germany).

**Determination of cell viability**

In order to test the viability of the cells synthesizing pathogenic peptides against repressed GAPDH expression, we used the Mossman assay [22]. Neuroblastoma SK-N-SH cells were placed into the wells of a 96-well plate, transfected with siRN A, and subsequently with the *Q103 *gene (as described above). Seventy-two hours after the onset of the experiment, the medium was removed from the wells. 90 μl of a fresh medium and 10 μl of a MTT solution (3-4,5-dimethylthiazol-2-yl-2,5- tetrazolium bromide, Sigma, USA), 5 mg/ml, in sterile PBS, were added to each well. The cells were incubated with MTT for 4 h at 37°С; then, the medium containing MTT was removed, and 100 μl of acidulated isopropanol (0.04 N HCl) was added into each well to dissolve blue formazane crystals in living cells. The signal was measured on a Fluorofot immunochemistry analyzer system (OOO “PROBANAUC HPRIBOR”, Russia) at 570 and 630 nm.

**Analysis of protein aggregation**

Protein aggregation was analyzed using two systems (*ex vivo *and *in vitro*). In the *ex vivo *system, neuroblastoma SK-N-SH cells were transfected with plasmids containing exon 1 of the huntington gene with a normal (Q25) and pathogenic (Q103) number of glutamine residues. After 8 h, when the amount of the mutant protein accumulated in the cells was sufficient for the analysis but no aggregates had yet been formed, the cells were lysed in a buffer with the following composition: 25 mM Tris-HCl pH 8.0, 20 mM NaCl, 1 mM EDТА. After the triple freeze-thaw cycle, the lysates were centrifuged at 10,000 *g*; the total protein concentration in the supernatant was measured using the Bradford protein assay [23]. The lysates were incubated at 37°С for 48 h and analyzed using the filter trap assay.

In the *in vitro *system, SK-N-SH neuroblastoma cells were transfected with plasmids encoding exon 1 of the* Q25 *and *Q103 *genes or with the *SOD1_wt_*and *SOD1_G93A_* genes. Twenty-four h after cell transfection with either* Q25 *or *Q103 *or 48 h after the transfection with *SOD1*, the cells were collected, washed thrice in cold PBS, and precipitated by centrifugation at 800 *g *for 5 min. A lysing buffer of composition 10 mM Tris-HCl pH 8.0, 150 mM NaCl, 2% SDS was added to the dry cellular precipitate. Following ultrasonic treatment for 1 min and incubation at 98°С for 2 min, the lysates were used to study the aggregate formation using the filter trap assay or the gel retardation assay.

**Filter trap assay**

The filter trap assay already described by Novoselova* et al*. [24] was employed in this study. The lysates of the transfected SK-N-SH neuroblastoma cells obtained in accordance with the above-described procedure were applied onto an acetate nitrocellulose membrane placed into a Dot-Blot apparatus (Hemel Hempstead, UK) connected to a vacuum pump. The membrane was washed with a buffer (10 mM Tris-HCl pH 8.0, 150 mM NaCl, 0.1% SDS) under pressure before and after application of the lysates. The presence of Q103 or SOD1 in the aggregates was determined using anti-EGFP antibodies.

**SDS PAGE electrophoresis and immunoblot assay**

In order to prepare specimens, the cells were collected, washed with cold PBS thrice, and centrifuged at 800 *g *for 5 min. A lysing buffer of composition 20 mM Tris-HCl рН 7.5, 150 mM NaCl, 0.5% Triton Х-100, 2 mM EDТА was added to the dry cellular precipitate. The lysates were centrifuged at 10,000 *g*; total protein concentration in the supernatants was measured using the Bradford protein assay. The amount of protein per specimen was 50 μg. After the electrophoresis, the proteins were transferred from the gel to a nitrocellulose membrane (Immobilon-P (PVDF), pore size 0.45 μm, Millipore Corporation, USA) using a TransBlot system (Bio-Rad, USA).

The zones of proteins of interest were detected using primary mono- or polyclonal antibodies and secondary antibodies against mouse or rabbit immunoglobulin conjugated to horseradish peroxidase. The peroxidase reaction was identified via enhanced chemiluminescence using the Chemidoc XRC visualization system (Bio-Rad, USA).

The immunoblot assay was conducted using anti- EGFP (Abcam, UK) and anti-GAPDH (Abcam, UK) monoclonal mouse antibodies; polyclonal rabbit antibodies against Hsp70 (R22) and tissue transglutaminase (Sigma, USA). Antibodies against mouse or rabbit immunoglobulins conjugated to horseradish peroxidase (Sigma, USA) were used as secondary antibodies.

**Modified SDS PAGE electrophoresis to analyze the SDS-insoluble cellular fraction (gel retardation assay)**

The modified SDS PAGE electrophoresis procedure was used to analyze the level of the proteins under study in the SDS-insoluble cellular fraction. This pro cedure involved the retardation of insoluble complexes in the stacking gel. The cellular precipitates were dissolved in a buffer with the following composition: 62.5 mM Tris-HCl pH 8.0, 2.5% SDS, 10% glycerol, 0.1 mM EDTA, 0.02 bromophenol blue. The specimens were subjected to ultrasonic treatment for 30 s and incubated at 98°C for 5 min.

The stacking gel had the following composition: 2% acrylamide, 0.15 bisacrylamide, 0.125 mM Tris-HCl pH 6.8, 0.1 SDS, 0.06% ammonium persulfate, and 0.06% N,N,N’,N’-tetramethylene diamine. The immunoblots were obtained for both the running and stacking gels.

## RESULTS AND DISCUSSION

**GAPH affects aggregation formation in cell models of Huntington’s disease**

**Fig. 1:**
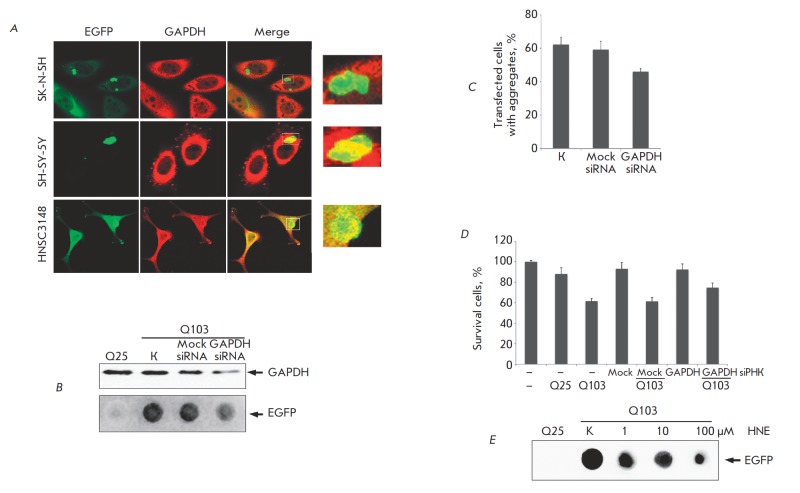
GAPDH affects the aggregation process in the cell model of Huntington’s disease. (a) GAPDH localizes to aggregates of mutant huntingtin. Human neuroblastoma SK-N-SH, SH-SY-5Y cells and HNSC3134 human embryo brain cells were transfected with the plasmid containing the sequence of 103 glutamines and was linked to the gene of the green fluorescent protein (EGFP). 48 h after the transfection, the cells were stained with anti-GAPDH antibody (2nd antibody is linked to a red fluorescent dye). Insert: yellow stain corresponds to the area of GAPDH and polyglutamine co-localization (488 nm and 543 nm channels). (b) Reduction in the GAPDH level attained with a specific siRNA proved by Western blotting (top panel) leads to downshifting of polyglutamine aggregation as revealed by a filter trap assay (bottom panel), (с) a decrease in the amount of aggregate-containing cells and (d) upregulation of the amount of surviving cells (data of the MTT assay) as compared with the control cells (Mock siRNA); (e) hydroxynonenal, HNE, specifically inactivating GAPDH reduces aggregation of polyglutamines in a filter trap assay

The functions of three proteins---GAPDH, tTG, and Hsp70---during the aggregation of mutant huntingtin (model of HD) and mutant SOD1 (model of ALS) were analyzed in this study. Three cell lines, SK-N-SH and SH-SY-5Y human neuroblastoma cells and HNSC3148 human embryonic brain cells, were used as the model of HD [25]. The cells were transfected with a plasmid containing exon 1 of the *Q103 *gene linked to the *EGFP* gene. Twelve h following the transfection, small bright spots emerged in the cells, which merged to form large fluorescent complexes over 100 nm in size during the subsequent 36 h (*Fig. 1A*). It should be mentioned that despite some time divergences, the patterns of aggregate formation were identical in all three types of cells. By using specific antibodies recognizing GAPDH, we demonstrated that the enzyme colocalized with aggregated polyglutamine chains (*Fig. 1A*).

It has been ascertained previously that GAPDH in SK-N-SH neuroblastoma cells is concentrated at the sites where oligomers, and subsequently large aggregates of mutant huntingtin, emerge [10]. Furthermore, this enzyme has been detected in deposits of mutant huntingtin in brain slices from patients with HD [26]. These data and the fact that this localization is observed in human embryonic cells indicate that this phenomenon is rather common and that GAPDH (whose fraction in the cellular protein can be as high as 2–4%) can affect aggregate formation during the development of HD.

The effect of GAPDH on the size of the growing aggregates of mutant huntingtin was assessed by reducing the level of this protein using siRN A. To this end, 24 h prior to launching the aggregation of mutant protein Q103, the SK-N-SH cells were transfected with the corresponding siRN A. After the reduction in the GAPDH level had been confirmed, the amount of aggregated material was determined. According to the immunoblot data, the use of this technology made it possible to reduce the amount of GAPDH in SK-N-SH human neuroblastoma cells by 50–60% (*Fig. 1B*). The filter trap assay was employed to study the aggregation process. This method allows one to determine the amount of aggregated material (with the polyglutamine repeat Q103 its major component (model of HD)) that remains on the acetate cellulose membrane after the cellular extracts obtained using SDS have passed through it. It can be clearly seen that a significant content of aggregates was detected in the control cell extract (the control cells had not been treated with siRN A) (*Fig. 1B*). The action of nonspecific siRN A (Mock siRN A) induced no changes in the aggregation process, whereas specific siRN A reduced the amount of aggregated SDSinsoluble mutant proteins (*Fig. 1B*). Moreover, it was demonstrated by counting the number of transfected cells with the aggregates and the diffusely distributed protein that the number of cells containing aggregates of the mutant protein decreased by 10% when enzyme synthesis was supressed (*Fig. 1C*).

It has been assumed that there is a direct relationship between the aggregation of mutant proteins and a reduction in viability for neuronal cells. Thus, we assessed how the suppression of GAPDH synthesis and, hence, a decrease in the aggregation level of the mutant protein affect the number of surviving cells. These experiments were conducted according to the procedure described above; however, cell viability was assessed using the Mossman assay 48 h after the transfection of siRN A and, subsequently, of the construct encoding the polyglutamine sequence (*Fig. 1D*). Indeed, the expression of mutant huntingtin was shown to reduce the number of viable cells by 40%. The preliminary transfection with a plasmid carrying the control siRN A does not affect the viability of both the control cells and those carrying Q103. The supression of GAPDH synthesis using specific siRN A resulted in a 18% increase in the number of survived cells expressing *Q103 *(as compared with the cells with a normal level of GAPDH) (*Fig. 1D*).

In order to demonstrate the significance of GAPDH as a pharmacological target, we searched for low-molecular- weight compounds that exhibit affinity with GAPDH among the published data and found several compounds, including hydroxynonenal (HNE ). HNE is known to be capable of reacting with cysteine and histidine residues in the enzyme molecule, thus inducing its inactivation [27]. HNE was introduced into the SK-N-SH human neuroblastoma cell culture expressing* Q103 *linked to the marker gene *EGFP*. The cell lysate was subsequently obtained and analyzed via a filter trap assay. The results of these experiments give grounds to consider that GAPDH is a target for small molecules: the aggregation degree of the complex of mutant huntingtin with GAPDH decreased by 45–50% under the effect of HNE at a pharmacologically relevant concentration (1 μM). This value is a fairly good therapeutic index; the aggregation degree decreased even more dramatically with increasing concentration of the compound (*Fig. 1E*). In our opinion, the effect of HNE is based on its ability to isolate some GAPDH molecules from its complex with mutant huntingtin; the aggregation process is supposed to be inhibited.

Thus, GAPDH localizes in pathogenic aggregates, along with mutant huntingtin, and seems to participate actively in the aggregation process at its early stages. This interpretation of the results is supported by the fact that a specific decrease in the amount of enzyme molecules accessible for aggregation, which can be attained using siRN A or a high-affinity compound, results in inhibition of the aggregation of the Q103– GAPDH complex.

**Investigation of the effect of tissue transglutaminase on the aggregation of mutant huntingtin**

**Fig. 2:**
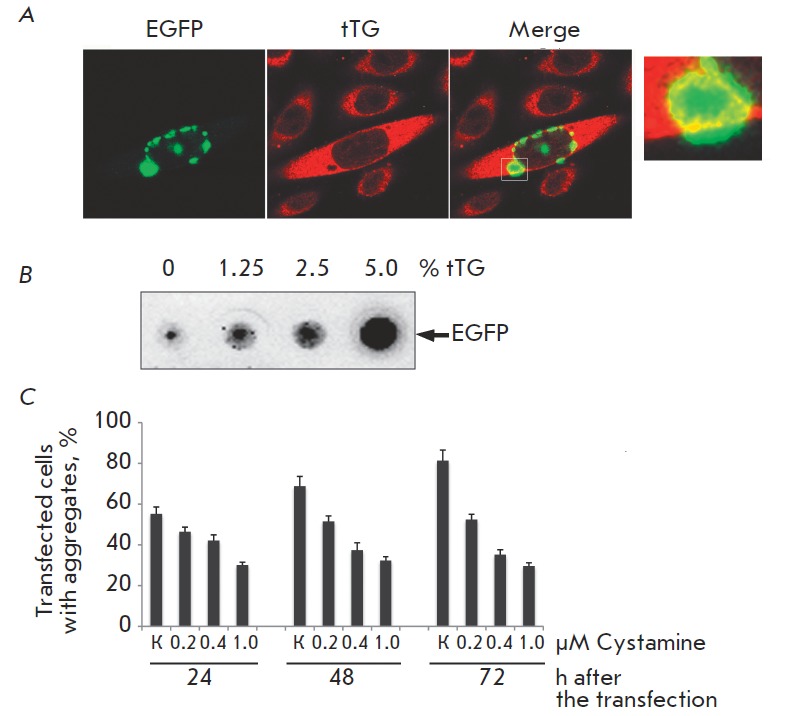
Tissue transglutaminase promotes the aggregation of mutant huntingtin. (а) tTG localizes to the aggregates of mutant huntingtin. Q103, conjugated with EGFP (green), tTG – red stain. Insert: co-localization of Q103 and tTG results in yellow staining; (b) addition of tTG to the extract of cells with polyglutamine causes an increase in the amount of aggregating material in a filter trap assay (the amount of tTG is given in percents of total protein); (с) inhibitor of tTG, cystamine, reduces the aggregation of polyglutamine in a dose-dependent fashion (the ordinates represent the number of cells with aggregates)

Aggregates of mutant huntingtin, ataxin, and some other pathogenic proteins can be formed via cross linking anomalously long polyglutamine chains with proteins that donate reactive lysines (in particular, GAPDH) in the tTG-catalyzed process [9, 28]. Immunofluorescence microscopy was used to determine the localization of tTG in cells that express *Q103 *in order to elucidate the role of this enzyme in our cell model. It turned out that tTG molecules are uniformly distributed over the cell cytoplasm, while enzyme clusters are observed around the Q103–GAPH aggregates. One can see that tTG colocalizes with mutant huntingtin (*Fig. 2A*). The participation of tTG in aggregate formation was proved by introducing the purified enzyme into an extract of cells transfected with the Q103–EGFP construct prior to the onset of aggregate formation. The *ex vivo *analysis of aggregation (see the EXPER IMENT AL section) conducted using the filter trap assay shows that the introduction of tTG dose-dependently increases the amount of Q103 that is SDS-insoluble and remains on the membrane (*Fig. 2B*).

To what extent does the inhibition of tTG activity affect the process of aggregate formation? In order to answer this question, we incubated SK-N-SH cells with the well-known enzyme inhibitor cystamine, right after the onset of aggregation (i.e., 5 h following the transfection with the *Q103–EGFP *gene). The counting of aggregate-containing cells demonstrated that the cystamine effect manifested itself 24 h after the start of the incubation; after a day, the effect of the inhibitor became more pronounced. Finally, cell-counting 3 days after the introduction of the inhibitor into the medium showed that cystamine at a concentration of 0.4 μM re duces by twofold the number of cells containing Q103– EGFP aggregates, while further increase in inhibitor concentration suppresses the aggregation to a greater extent (*Fig. 2C*). Interestingly, when using cystamine at a concentration of 1 μM, the fraction of aggregatecontaining cells remains constant with time and, in our case, was equal to 25–28% of the total number of transfected cells. In the untreated population, the fraction of aggregate-containing cells increased and was equal to 82% 72 h after the transfection. This fact can be an indication that the formation of polyglutamine aggregates is caused not only by the action of tTG, but also by another mechanism (e.g., that in the “polar zipper” model) [3].

**Participation of GAPDH and tTG in aggregate formation in mutant SOD1**

**Fig. 3:**
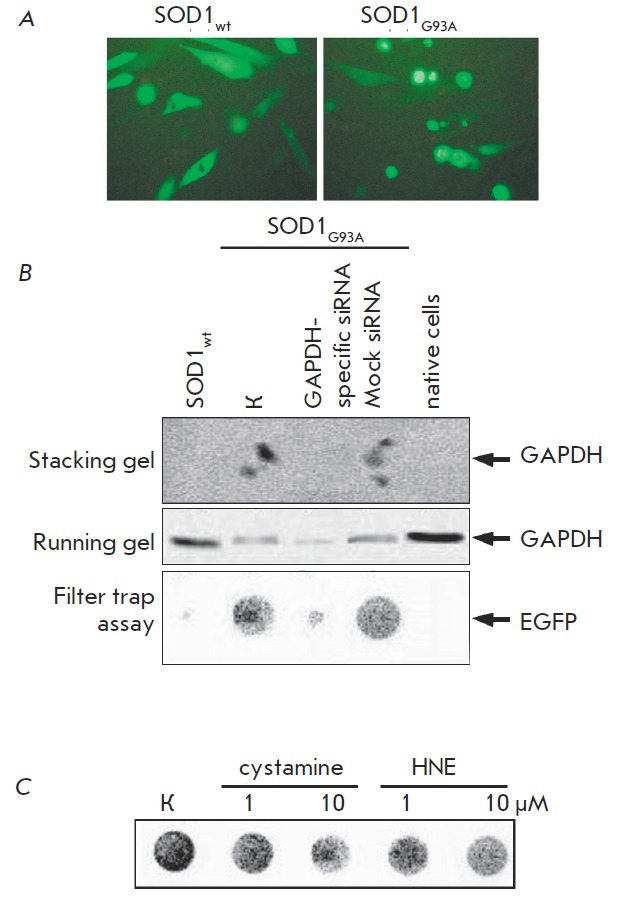
GAPDH participates in the formation of aggregates in a cell model of amyotrophic lateral sclerosis (ALS). (a) SK-N-SH human neuroblastoma cells 48 h after the transfection with the gene of mutant SOD1 (G93A) or wild-type SOD1, conjugated with the gene of a green fluorescent protein (EGFP). Right panel: SOD1-G93A forms insoluble aggregate structures; (b) application of GAPDH-specific siRNA prevents aggregation of mutant SOD1 (data of gel shift assay, two top panels and data of filter trap assay, bottom panel); (с) incubation of ALS-imitating cells with cystamine and HNE down-regulates SOD1 aggregation (filter trap assay)

Assuming that GAPDH and tTG promote the aggregation not only of huntingtin but of other mutant proteins as well, we analyzed the functions of these enzymes in aggregate formation by the example of the cellular model of ALS. To this end, plasmids carrying the* SOD1_G93A_*and *SOD1_wt_*genes linked to the green fluorescent protein gene were used. A microscopic analysis of SK-N-SH neuroblastoma cells transfected with these plasmids demonstrated that the mutant SOD1_G93A_, unlike SOD1_wt_, can form aggregates in 36–48 h (*Fig. 3A*).

Does GAPDH play a role in the formation of aggregates of mutant SOD1 as important as that in the model of HD? In order to answer this question, we used the technology of specific small interfering RN As. Lysates of SK-N-SH cells simultaneously transfected with specific or control siRN A and *SOD1_wt_*or *SOD1_G93A_*were analyzed by the gel retardation assay and immunoblot assay and using the filter trap assay (*Fig. 3B*).

As follows from the electrophoresis data, the content of GAPDH that can penetrate the running gel in the lysate of the cells treated with specific siRN A is considerably lower than that in the lysates of the control (intact) cells and cells carrying SOD1_wt_ (*Fig. 3B, *middle panel). Both in the control cells and in the cells transfected with Mock siRN A (both cell types carrying mutant SOD1), the level of GAPDH that can penetrate the running gel is reduced. However, it is the lysates obtained from these cells that contain a significant amount of the aggregates remaining in the stacking gel (*Fig. 3B*, top panel). These results have also been supported in an experiment employing the filter trap assay. Aggregates of mutant SOD1 (presumably containing GAPDH) were detected in the lysates of these cells (*Fig. 3B*, bottom panel).

In addition to GAPDH-specific siRN A, HNE was also used to repress GAPDH. We demonstrated using the filter trap assay that HNE at a concentration of 1 μM represses the aggregation of mutant SOD1; an increase in HNE concentration to 10 μM strengthened this effect (*Fig. 3C*). The effect of HNE can be attributed to the fact that the formation of free radicals is increased in patients with ALS, as well as in those with numerous other pathological processes, while the oxidative stress disrupts the GAPDH structure. The regions of the enzyme molecule are exposed and bind to mutant proteins, giving rise to large complexes [29]. We hy pothesize that HNE impedes the formation of these complexes; i.e., it inhibits SOD1 aggregation.

The participation of tTG in the formation of SOD1– GAPDH aggregates has also been demonstrated using inhibitory analysis. We used cystamine to ascertain that the suppression of tTG activity reduces the weight of the aggregating material on a filter. However, this effect can be achieved at high cystamine concentrations (at least 10 μM) exceeding pharmacological values (*Fig. 3C*). It is harder to interpret the fact of suppression of aggregation of mutant SOD1 when using cystamine. It is possible that he inhibition with tTG prevents the formation of covalent bonds both between GAPDH molecules and between GAPDH molecules and other proteins.

**Hsp70 chaperon represses the aggregation of mutant huntingtin in the cellular model of HD**

**Fig. 4:**
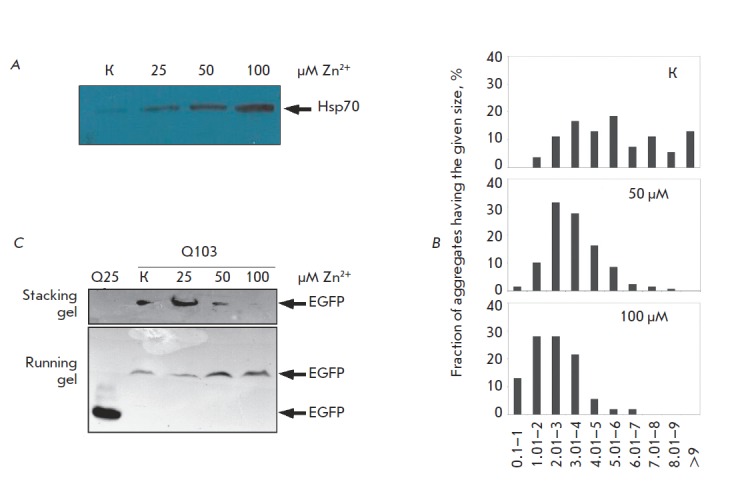
Hsp70 prevents aggregation of mutant huntingtin in a cell model of HD. (a) Expression of Hsp70 gene controlled by the metallothionein promoter in SK-N-SH cells is dose-dependently induced by zinc; (b) histograms demonstrating the distribution of the Q103 aggregate size dependent on the zinc concentration and, accordingly, the level of Hsp70 expression; (c) the stepwise increase in Hsp70 content in SK-N-SH neuroblastoma cells transfected with Q103 leads to a reduction in polyglutamine aggregation in a filter trap assay

Hsp70 chaperon plays a significant role in preventing complex formation between damaged or mutant polypeptides [30]. The effect of Hsp70 on the aggregation of mutant huntingtin was studied using SK-N-SH neuroblastoma cells transfected with the *Hsp70 *gene under the control of an inducible metallothionein promoter as a model. *Hsp70 *expression was induced using a zinc salt (ZnSO_4_), which can be used to increase the protein level in a dose-dependent manner (*Fig. 4A*), 6 h prior to the transfection of SK-N-SH cells with a plasmid carrying the *Q103 *gene. The diameter of the aggregates of mutant huntingtin was determined 48 h after the transfection. The mean diameter of the aggregates in the transfected cells treated with 50 μM ZnSO_4_ was 3.15 ± 0.69 μm, while in the control cells it was equal to 6.82 ± 0.98 μm. A further decrease both in the number of aggregate-carrying cells and aggregate size (the mean diameter being equal to 1.52 ± 0.19 μm*, Fig. 4B*) was observed as the ZnSO_4_ concentration was increased to 100 μM. The effect of Hsp70 on the amount of aggregating material was analyzed using the filter trap assay. It turned out that an increase in ZnSO_4_ concentration and, hence, in the Hsp70 level results in a decrease in the amount of material containing the EGFP marker retained on the filter surface (*Fig. 4C*).

It has been known that molecular chaperons, in particular Hsp70, participate in the prevention of aggregate formation in pathogenic or damaged proteins; however, the mechanisms underlying this effect remain unclear. In this context, data indicating that the chaperon forms a complex with the aggregating monoand oligomers of mutant huntingtin are of significant interest [31]. This complex is of a dynamic nature. It is assumed that Hsp70 impedes the incorporation of polyglutamine molecules into the aggregates of mutant chromatin being formed. However, the results of a recently published study [10] indicate that Hsp70 affects not only mutant huntingtin, but also GAPDH molecules, which significantly enhance the aggregation. Based on the results of this study an assumption can be made that at least in the cell models of HD, Hsp70 is capable of impeding the formation of the polyglutamine– GAPDH complex, thus protecting the enzyme against the cross-linking effect of tTG. This hypothesis does not contradict the theories of the function of chaperons in protecting cells against neuropathogenic stimuli. However, it undoubtedly requires thorough verification.

## CONCLUSIONS

It has been ascertained in this study using cell models of Huntington’s disease and amyotrophic lateral sclerosis that two enzymes–glyceraldehyde-3-phosphatedehydrogenase and tissue transglutaminase–play a significant role, along with the pathogenic proteins specific to these disorders. The former enzyme participates in complex formation with pathogenic proteins in both models of the diseases; its blockage reduces the aggregation rate. Transglutaminase presumably catalyzes the formation of the GAPDH complex with pathogenic cellular proteins. Hsp70 chaperon is the factor that prevents the aggregation; an increase in its expression reduces the pathogenic symptoms in a dose-dependent fashion. The data obtained provide ground to regard all three proteins as promising pharmacological targets.

## Abbreviations

EGFP: enhanced green fluorescence protein
ALS: amyotrophic lateral sclerosis
HSP: heat shock protein
HD: Huntington’s disease
GAPDH: glyceraldehyde-3-phosphate dehydrogenase
HNE: hydroxynonenal
SDS: sodium dodecyl sulfate
PAAG: polyacrylamide gel
SOD: superoxide dismutase
tTG: tissue transglutaminase
PBS: phosphate buffer saline
